# A Comparative Evaluation of the Effects of Three Different Mouthwashes on Salivary pH in Patients Undergoing Fixed Orthodontic Treatment: A Randomized Clinical Trial

**DOI:** 10.7759/cureus.116931

**Published:** 2026-09-25

**Authors:** Sruthi Jeevagan, Sathi Rami Reddy Mora, Mohamed Rahmankhan N., V. Vijaykumar, Santhosh Kumar, Santha Kumari

**Affiliations:** 1 Department of Orthodontics and Dentofacial Orthopaedics, Adhiparasakthi Dental College and Hospital, Melmaruvathur, IND

**Keywords:** chlorhexidine mouth wash, fixed orthodontic treatment, hetafu mouthwash, listerine, salivary ph

## Abstract

Introduction

Orthodontic appliances have been reported to influence salivary composition and properties, including alterations in pH and buffering capacity, which may increase the vulnerability of patients to caries development. In addition to mechanical plaque control, mouthwashes and other chemical agents are frequently advised to improve oral hygiene in orthodontic patients. This randomized clinical experiment assesses and compares the effects of three distinct mouthwashes on the salivary pH level in patients receiving fixed orthodontic therapy.

Methods

This randomized parallel-group single-blind clinical trial involved 120 patients who attended fixed orthodontic treatment, randomly divided into three groups (n = 40 each): Hetafu CUTE, chlorhexidine, and Listerine mouthwash users. Every participant received two 10-15 mL rinses per day, each of which lasted 30-60 seconds. Saliva pH was measured using standard pH paper at baseline, directly after mouthwash use (five minutes), and one week after the beginning of the intervention. The results were analyzed using a mixed-design repeated-measures analysis of variance (ANOVA) with Greenhouse-Geisser correction, and Bonferroni-adjusted post-hoc comparisons were performed where appropriate. Statistical significance was set at p < 0.05.

Results

A total of 120 participants were included, with 40 in each group. Baseline salivary pH was comparable among the groups (p = 0.157). At five minutes, salivary pH was higher with Hetafu CUTE (7.75 ± 0.78) than with chlorhexidine (7.08 ± 1.00) and Listerine (7.15 ± 1.37) (p = 0.014). At one week, pH increased with Hetafu CUTE (7.93 ± 0.69) but decreased with chlorhexidine (6.78 ± 0.80) and Listerine (6.70 ± 1.34) (p < 0.001). The group × time interaction was statistically significant (p < 0.001), with Bonferroni-adjusted comparisons showing higher pH with Hetafu CUTE than chlorhexidine (p = 0.006) and Listerine (p = 0.023).

Conclusion

Within the scope of the study, the Hetafu CUTE mouth rinse showed a more pronounced and prolonged increase in saliva pH compared to Listerine and chlorhexidine. This characteristic may contribute to the maintenance of oral pH homeostasis and prevention of enamel demineralization and white spot lesions. However, further research, including large-scale randomized controlled trials, is warranted to validate these findings and establish the clinical efficacy of Hetafu CUTE.

## Introduction

Fixed orthodontic treatment is commonly used to treat malocclusion and improve functional efficiency and facial esthetics. Orthodontic attachments such as brackets, bands, and archwires are advantageous but can promote plaque retention by creating areas that are difficult to clean, predisposing patients to enamel demineralization, gingival inflammation, and dental caries during treatment [1]. In these complications, white spot lesions (WSLs) are early and clinically detectable signs of demineralization of the enamel. The prevalence of WSLs among orthodontic patients varies widely [2,3]. These lesions can be formed shortly after appliances are introduced as a result of increased plaque accumulation and a shift in the bacterial composition of the plaque toward acidogenic and aciduric organisms such as *Streptococcus mutans* and *Lactobacillus* spp. [3].

The protective role of saliva in terms of buffering capacity, antimicrobial activity, and remineralization capability should also be emphasized. The balance between demineralization and remineralization is controlled by the physiologic variables of saliva that dictate the oral pH [1]. A reduction in salivary pH enhances the risk of mineral loss from enamel, whereas a neutral or alkaline environment favors remineralization and inhibits cariogenic activity. Orthodontic appliances have been reported to influence salivary composition and properties, including alterations in pH and buffering capacity, which may increase the vulnerability of patients to caries development [1,4]. Furthermore, the associated increase in plaque accumulation and microbial changes further exacerbates this imbalance [5].

Besides mechanical plaque management, chemical agents like mouthwashes are often recommended to enhance oral cleanliness in orthodontic patients. Various formulations incorporating antimicrobial substances, fluoride, or botanical components have shown advantageous effects in managing plaque and altering the oral milieu, including salivary pH [6]. However, the influence of different mouthrinses on the pH of saliva of patients with a fixed orthodontic appliance has not been compared. Therefore, the purpose of our research is to compare the influence of three different mouthrinses on the pH level of saliva of patients with a fixed orthodontic appliance. For the purpose of obtaining the correct result and reducing the bias, a randomized clinical trial design was used.

## Materials and methods

This study was designed as a randomized, parallel-group clinical trial in accordance with the Consolidated Standards of Reporting Trials (CONSORT) guidelines to comparatively evaluate the effects of three different mouthwashes on salivary pH in patients undergoing fixed orthodontic treatment. The study was approved by the Adhiparasakthi Dental College and Hospital's Institutional Ethics Committee (IEC) (Approval No.: ECR/1742/APDCH/ORTHO/FM01/TN2025). The study was registered with the Clinical Trials Registry of India (CTRI/2026/02/103317). It was conducted among 120 patients who met the inclusion criteria from February to April 2026, in the Department of Orthodontics, Adhiparasakthi Dental College and Hospital, Melmaruvathur. The sample size was determined by considering the previous study’s power analysis, which explained the rationale for the saliva pH difference between the groups. With 80% power and a 95% confidence level, the population was divided into three equal groups.

The inclusion criteria were as follows: patients who received standard orthodontic treatment and were aged between 12 and 30 were recruited. In addition, only those who were healthy and willing to participate in the study were included. Furthermore, those who did not have caries or periodontal disease and had good oral hygiene were recruited. However, a significant number of the patients were excluded from the study for specific reasons. Those patients had conditions that could affect saliva flow, or they were on medication that could influence saliva’s composition. Exclusion criteria also included simultaneous antibiotic use or mouth rinse use within two weeks after the treatment, hypersensitivity to any of the mouthwash’s ingredients, failure to meet the requirements, and unwillingness to cooperate.

Following the selection process, the participants were divided into three intervention groups based on a computer-generated randomization scheme. Each of the three intervention groups comprised n = 40 participants. Allocation concealment was achieved by placing the assignment in an envelope with a concealed label. The experiment was performed as a single-blind trial where the assessor who measured the salivary pH was blind to the process.

Group A participants were administered Hetafu CUTE mouthwash (Lasarkaali Life Sciences Pvt. Ltd., Hyderabad, India), which is a commercially marketed edible mouthwash described by the manufacturer as a probiotic formulation and containing botanical ingredients such as *Azadirachta indica* extract, *Salvadora persica* extract, *Syzygium aromaticum* extract, *Mentha piperita* extract, and *Terminalia chebula* extract as active ingredients. These botanical ingredients have been reported to possess antimicrobial, anti-inflammatory, antioxidant, and plaque-reducing effects. This mouthwash product also contains a high percentage of inactive ingredients with an approximate pH of 7.62, making it an alkaline solution. As the present study did not undertake microbiological characterization of the product, specific probiotic strains and viable counts, expressed as colony-forming units (CFU), were not independently determined.

Group B received a chlorhexidine-containing mouthwash, with chlorhexidine gluconate as the active ingredient. The inactive ingredients included water, alcohol (in some formulations), glycerin, PEG-40 sorbitan diisostearate, sodium saccharin, flavoring agents, and coloring agents. Chlorhexidine is a substantive antiseptic with a broad spectrum of antimicrobial activity and belongs to the class of weakly acidic antimicrobial agents, with a pH range of approximately 5-7.

Group C participants received Listerine antiseptic mouthwash (Johnson & Johnson Consumer Inc., Skillman, NJ, USA) containing eucalyptol, menthol, methyl salicylate, and thymol as active ingredients. The inactive ingredients included coloring agents, water, sorbitol, Poloxamer 407, benzoic acid, and flavoring agents. The product also contains a high percentage of alcohol close to 21.6% and is associated with a lower PH range of 4.3-4.88, thus strongly acidic in nature (Figure 1).

**Figure 1:**
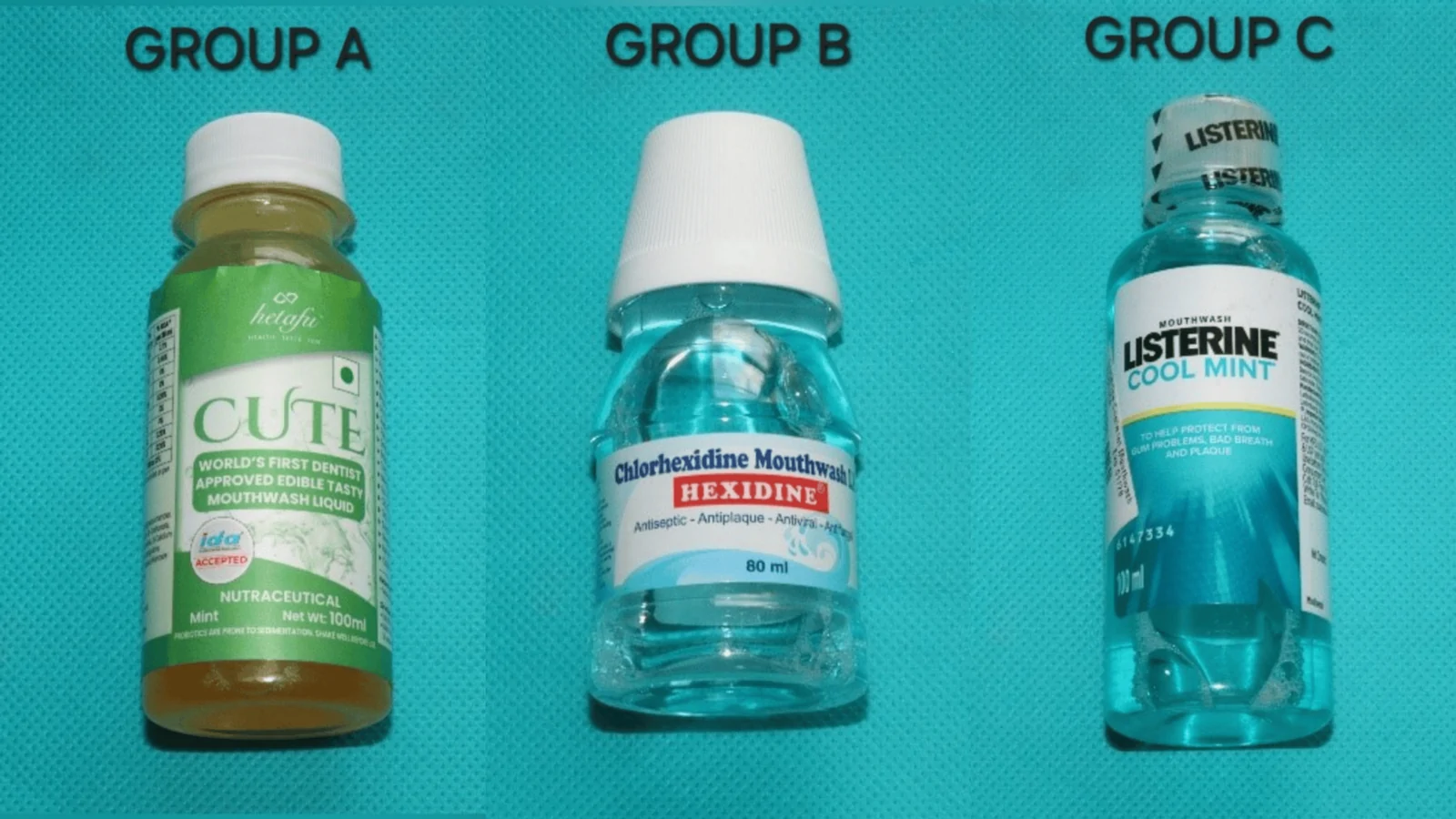
The three mouthwashes used in the study. Group A - Hetafu CUTE mouthwash; Group B - Chlorhexidine mouthwash; Group C - Listerine antiseptic mouthwash

All participants were instructed to use 10-15 mL of the selected mouthwash as part of their after-brushing oral hygiene routine, twice a day for 30-60 seconds. In addition, all participants received oral hygiene instructions to standardize the oral hygiene status throughout the study period. As the primary outcome, the pH of saliva was measured using standardized protocols for all participants. In particular, the unstimulated saliva pH was estimated three times: before the trial (baseline), five minutes after the mouthwash use to determine immediate effects, and a week after the beginning of the trial to assess short-term effects. At the one-week assessment, unstimulated saliva was collected before the participant's scheduled morning mouthwash use, thereby avoiding immediate contamination by the mouthwash formulation. Salivary pH was assessed using full-range pH (1-14) indicator strips (MCP Fisher Scientific, Waltham, MA, USA). The color developed on the strip was visually compared with the reference color chart supplied with the product under consistent lighting conditions. Finally, all 120 participants completed the trial, and the results were analyzed using an intention-to-treat approach (Figures 2, 3).

**Figure 2:**
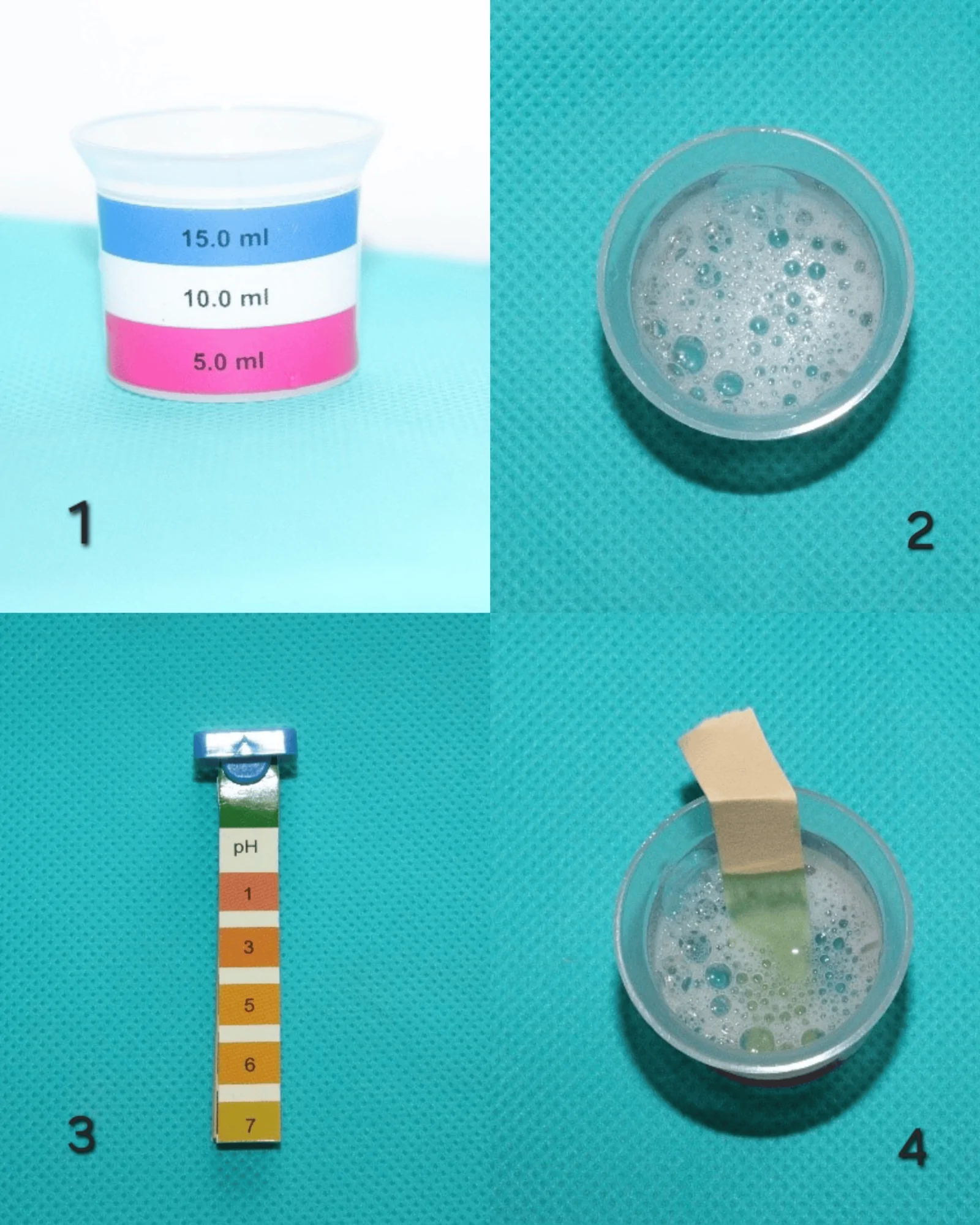
Stepwise procedure for the estimation of pH using a universal pH indicator strip. (1) Collection of the sample, (2) preparation of the sample for testing, (3) universal pH indicator strip, and (4) immersion of the pH strip in the sample for pH assessment.

**Figure 3:**
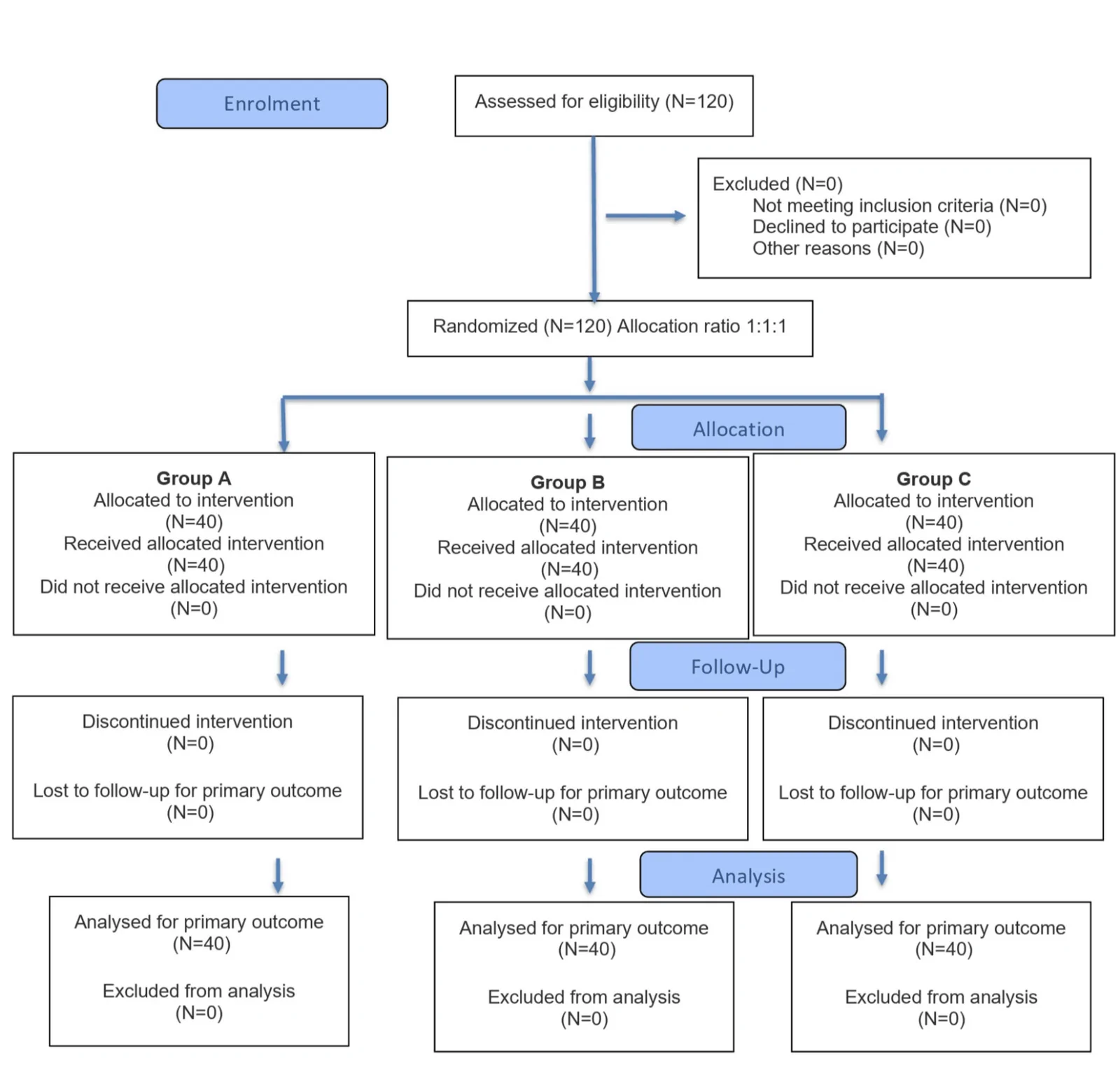
CONSORT flow diagram of participant recruitment, allocation, follow-up, and analysis. CONSORT: Consolidated Standards of Reporting Trials

Data were analyzed using IBM SPSS Statistics for Windows, Version 26 (Released 2018; IBM Corp., Armonk, New York, United States). Descriptive statistics were expressed as mean ± standard deviation (SD). A mixed-design repeated-measures analysis of variance (ANOVA) was performed to evaluate changes in salivary pH over time, with mouthwash group (Hetafu CUTE, chlorhexidine, and Listerine) as the between-subjects factor and assessment time (baseline, five minutes, and one week) as the within-subjects factor. The mouthwash group × time interaction was evaluated as the primary outcome to determine whether the pattern of salivary pH change differed among the three groups. Mauchly’s test of sphericity was performed, and the Greenhouse-Geisser correction was applied when the assumption of sphericity was violated. Partial eta squared (ηp^2^) was reported as the measure of effect size. Where significant effects were identified, time-specific between-group comparisons and Bonferroni-adjusted pairwise comparisons were performed as follow-up analyses. Baseline-adjusted longitudinal changes were additionally evaluated using a linear mixed model, with baseline salivary pH included as a covariate. Estimated marginal means were calculated and graphically presented to illustrate changes in salivary pH over time. A two-sided p-value <0.05 was considered statistically significant.

## Results

In this study, 120 patients were recruited and divided into three groups: 40 patients were assigned to the Hetafu CUTE group, 40 patients were assigned to the chlorhexidine group, and 40 patients were assigned to the Listerine group. Every participant utilized only the mouthwash assigned to their specific study group. At baseline (T0), the mean salivary pH was comparable across the three groups: Hetafu CUTE (7.18 ± 0.78), chlorhexidine (7.30 ± 0.97), and Listerine (7.55 ± 0.55). The overall baseline mean was 7.34 ± 0.79, indicating a near-neutral salivary environment across participants prior to intervention. At five minutes post-rinse, the Hetafu CUTE group demonstrated an increase in mean salivary pH (7.75 ± 0.78), whereas chlorhexidine (7.08 ± 1.00) and Listerine (7.15 ± 1.37) showed comparatively lower values. At one week, the Hetafu CUTE group exhibited a further increase in pH (7.93 ± 0.69), while chlorhexidine (6.78 ± 0.80) and Listerine (6.70 ± 1.34) showed a reduction compared to baseline (Table 1).

**Table 1:** Mean salivary pH at different time intervals among study groups (n = 120).

Time Point	Hetafu CUTE (n = 40)	Chlorhexidine (n = 40)	Listerine (n = 40)	Total (n = 120)
T0 (baseline)	7.18 ± 0.78	7.30 ± 0.97	7.55 ± 0.55	7.34 ± 0.79
Five minutes	7.75 ± 0.78	7.08 ± 1.00	7.15 ± 1.37	7.33 ± 1.11
One week	7.93 ± 0.69	6.78 ± 0.80	6.70 ± 1.34	7.13 ± 1.13

Mauchly’s test of sphericity indicated that the assumption of sphericity was violated for the time effect (W = 0.944, χ^2^(2) = 6.681, p = 0.035). The Greenhouse-Geisser epsilon was 0.947, so corrected degrees of freedom were used for the within-subject time effect and the group × time interaction. This adjustment accounts for the violation of the sphericity assumption and provides an appropriate correction for the repeated-measures ANOVA results (Table 2).

**Table 2:** Mauchly’s test of sphericity for time. Sphericity was violated; Greenhouse-Geisser-corrected tests are therefore reported.

Within-subject effect	Mauchly’s W	χ^2^	df	p-value	Greenhouse-Geisser ε
Time	0.944	6.681	2	0.035	0.947

Mixed-design repeated-measures ANOVA demonstrated a statistically significant main effect of time on salivary pH, F(1.894, 221.597) = 3.802, p = 0.026, partial η^2^ = 0.031. A statistically significant main effect of mouthwash group was also observed, F(2, 117) = 5.904, p = 0.004, partial η^2^ = 0.092. Importantly, a statistically significant mouthwash group × time interaction was observed, F(3.788, 221.597) = 17.205, p < 0.001, partial η^2^ = 0.227, indicating that changes in salivary pH over time differed significantly among the three mouthwash groups (Table 3).

**Table 3:** Mixed-design repeated-measures ANOVA of salivary pH according to mouthwash group and time. ^†^ Greenhouse-Geisser corrected. For the between-subject group effect, uncorrected between-subject df are shown. ANOVA: analysis of variance

Effect	F	df	p-value	Partial η^2^
Time^†^	3.802	1.894, 221.597	0.026	0.031
Mouthwash group	5.904	2, 117	0.004	0.092
Mouthwash group × time^†^	17.205	3.788, 221.597	<0.001	0.227

Between-group comparisons at each assessment time point showed no statistically significant difference in salivary pH at baseline, F(2,117) = 2.37, p = 0.098, indicating comparable baseline values among the three groups. A statistically significant between-group difference was observed at five minutes, F(2,117) = 4.73, p = 0.011, and this difference was more pronounced at one week, F(2,117) = 19.33, p < 0.001 (Table 4).

**Table 4:** Between-group comparison of salivary pH at each assessment time. Time-specific one-way comparisons based on the three group distributions at each assessment.

Time point	F	df	p-value	Partial η^2^
Baseline	2.37	2, 117	0.098	0.039
Five minutes	4.73	2, 117	0.011	0.075
One week	19.33	2, 117	<0.001	0.249

A baseline-adjusted linear mixed-model analysis was performed for salivary pH at five minutes and one week, with baseline salivary pH included as a covariate. After adjustment for baseline pH, significant effects of mouthwash group, F(2,115.997) = 22.021, p < 0.001, and time, F(1,117) = 6.606, p = 0.011, were observed. Baseline salivary pH was also a significant covariate, F(1,116) = 39.666, p < 0.001. The mouthwash group × time interaction remained statistically significant, F(2,117) = 6.381, p = 0.002, indicating that changes in salivary pH between five minutes and one week differed among the three mouthwash groups (Table 5).

**Table 5:** Baseline-adjusted mixed-model analysis of salivary pH at five minutes and one week. A linear mixed model was fitted to post-intervention salivary pH measurements at five minutes and one week, with baseline salivary pH included as a covariate. The model used restricted maximum likelihood estimation, Satterthwaite degrees of freedom, a subject-level random intercept, and an unstructured covariance structure for repeated measurements.

Fixed effect	Numerator df	Denominator df	F	p-value
Mouthwash group	2	115.997	22.021	<0.001
Time	1	117	6.606	0.011
Baseline salivary pH	1	116.000	39.666	<0.001
Mouthwash group x time	2	117	6.381	0.002

Baseline-adjusted estimated marginal means demonstrated different patterns of salivary pH change among the three mouthwash groups. At five minutes, the adjusted mean salivary pH was 7.852 for Hetafu CUTE, 7.100 for chlorhexidine, and 7.023 for Listerine. At one week, the corresponding adjusted means were 8.027, 6.800, and 6.573, respectively. Thus, the adjusted mean increased from five minutes to one week in the Hetafu CUTE group, whereas decreases were observed in both the chlorhexidine and Listerine groups. This pattern was consistent with the statistically significant mouthwash group × time interaction in the baseline-adjusted mixed model (F(2,117) = 6.381, p = 0.002) (Table 6).

**Table 6:** Baseline-adjusted estimated marginal means of salivary pH by mouthwash group and time. Estimated marginal means are adjusted for baseline salivary pH = 7.34.

Mouthwash group	Time	Adjusted mean	SE	95% CI
Hetafu CUTE	Five minutes	7.852	0.367	4.950-10.754
Hetafu CUTE	One week	8.027	0.368	5.420-10.633
Chlorhexidine	Five minutes	7.100	0.367	4.070-10.131
Chlorhexidine	One week	6.800	0.367	4.081-9.520
Listerine	Five minutes	7.023	0.367	4.195-9.851
Listerine	One week	6.573	0.368	4.031-9.114

The overall baseline-adjusted estimated marginal means of salivary pH, averaged across the two post-intervention assessment times, were 7.939 for the Hetafu CUTE group, 6.950 for the chlorhexidine group, and 6.798 for the Listerine group (Table 7).

**Table 7:** Overall baseline-adjusted marginal means. Marginal means are adjusted for baseline salivary pH = 7.34 and averaged across the two post-intervention time points.

Mouthwash group	Adjusted mean
Hetafu CUTE	7.939
Chlorhexidine	6.950
Listerine	6.798

Baseline-adjusted pairwise comparisons of the three mouthwash groups are presented. After adjustment for baseline salivary pH and averaging across the two post-intervention assessment times, the estimated marginal mean salivary pH was 7.939 for Hetafu CUTE, 6.950 for chlorhexidine, and 6.798 for Listerine. Bonferroni-adjusted pairwise comparisons showed that the adjusted mean salivary pH was significantly higher with Hetafu CUTE than with chlorhexidine (mean difference = 0.989, 95% CI: 0.540-1.437, p < 0.001) and Listerine (mean difference = 1.142, 95% CI: 0.685-1.598, p < 0.001). No statistically significant difference was observed between chlorhexidine and Listerine (mean difference = 0.153, 95% CI: -0.299 to 0.604, p = 1.000) (Table 8).

**Table 8:** Pairwise group comparisons. Pairwise comparisons were Bonferroni-adjusted.

Comparison	Adjusted mean difference	95% CI	Bonferroni-adjusted p-value
Hetafu CUTE vs chlorhexidine	0.989	0.540 to 1.437	<0.001
Hetafu CUTE vs Listerine	1.142	0.685 to 1.598	<0.001
Chlorhexidine vs Listerine	0.153	-0.299 to 0.604	1.000

The estimated marginal means demonstrated different patterns of salivary pH change across the three mouthwash groups, consistent with the statistically significant mouthwash group × time interaction observed in the repeated-measures analysis. The Hetafu CUTE group showed an increasing trend in salivary pH over time, whereas the chlorhexidine and Listerine groups showed decreasing trends (Figure 4).

**Figure 4:**
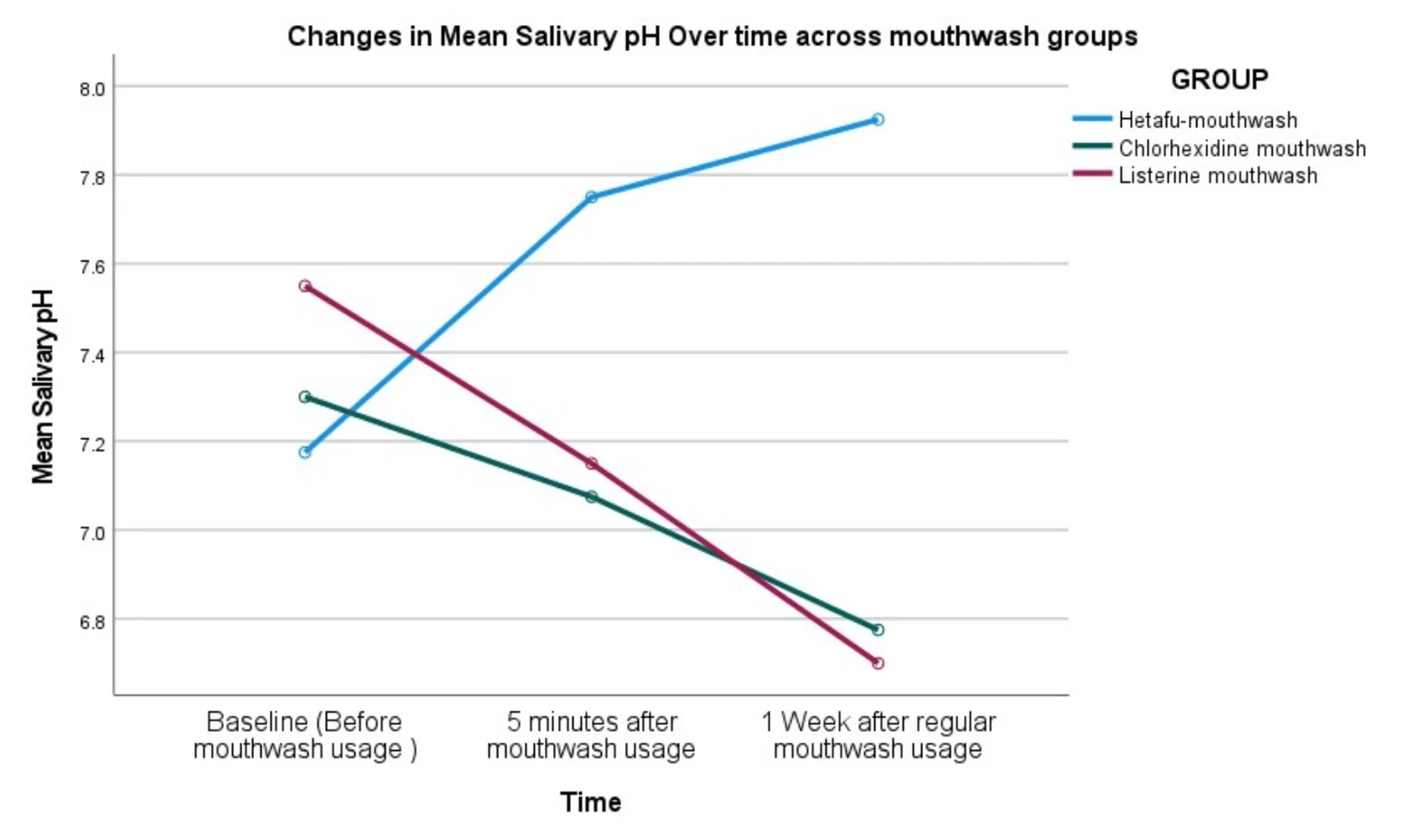
Changes in estimated marginal mean salivary pH across assessment time points according to mouthwash group.

## Discussion

Fixed orthodontic appliances create plaque-retentive niches that favor microbial accumulation and may contribute to an oral environment conducive to enamel demineralization and the development of WSLs [1-5,7]. Saliva plays an important protective role in maintaining oral homeostasis through its buffering properties and its ability to neutralize acids [1,4]. Therefore, interventions that help maintain a favorable oral environment may be of particular relevance in patients undergoing fixed orthodontic treatment.

Chlorhexidine is widely recognized as an effective antimicrobial agent because of its broad-spectrum activity and substantivity, and its established clinical benefits are primarily related to plaque control and gingival health [8,9]. Essential oil-based mouthwashes have been extensively investigated for their antimicrobial, antiplaque, and anti-gingivitis properties [10-12]. However, the available evidence from these studies predominantly concerns plaque and gingival outcomes rather than direct changes in salivary pH.

Probiotics have gained increasing attention as biologically based adjuncts for maintaining oral health. Previous systematic evidence has evaluated their potential role in the management of dental caries and periodontal diseases [13]. Available clinical evidence also supports the potential role of probiotic interventions in oral health management among orthodontic patients. A randomized clinical trial comparing probiotic and chlorhexidine oral rinses evaluated plaque-inhibitory and antimicrobial outcomes in orthodontic patients [14]. Systematic evidence has further examined the effects of probiotics on clinical and microbial outcomes during orthodontic treatment [15]. More recent clinical research comparing different preventive mouthwash formulations reflects the growing interest in biologically based approaches to oral health management in orthodontic patients [16]. Previous investigations involving Hetafu-based products have reported beneficial effects on selected oral microbial and clinical parameters [17-19]. However, these findings should be distinguished from direct evidence regarding changes in salivary pH, as evidence specifically addressing the effect of probiotic interventions on salivary pH remains limited. Although Hetafu CUTE is commercially marketed as an edible probiotic mouthwash, the present study did not independently characterize the probiotic component of the formulation, including specific strains or viable counts. Therefore, the present study was undertaken to compare the effect of Hetafu CUTE mouthwash, a commercially marketed edible probiotic mouthwash, with chlorhexidine and Listerine on salivary pH in patients undergoing fixed orthodontic treatment.

Under resting conditions, unstimulated saliva is continuously secreted while playing an important role in the maintenance of the health and stability of the oral cavity. It aids in the maintenance of oral moisture, lubrication of the oral mucosa, and provides a protective environment against dental caries. Saliva properties like pH are among the variables that contribute to the maintenance of a healthy oral cavity and a proper remineralization/demineralization balance. For instance, in their study, Preethi et al. (2010) evaluated unstimulated salivary pH of caries-free and caries-active children and found that saliva pH was significantly lower for children with caries [20]. This emphasizes the importance of salivary pH to the health of the oral cavity and its susceptibility to caries. Hence, measuring unstimulated salivary pH would offer significant insight into changes in the oral environment and may be extremely relevant in cases of fixed orthodontic treatment, where the changes in the oral environment will influence the caries risk. Therefore, unstimulated saliva was utilized for the evaluation of salivary pH in the present study, as it reflects the basal salivary environment under resting conditions and is continuously present in the oral cavity.

Furthermore, in their quasi-experimental study, Arsha Darsh et al. assessed salivary pH and salivation rate among patients who had fixed orthodontic braces and found that their salivary pH significantly decreased six months after receiving the treatment, whereas their salivation rate increased [21]. This finding implies that fixed orthodontic appliances could affect the pH of the saliva of orthodontic patients, which, in turn, supports the need to monitor the salivary pH of such patients throughout the treatment.

Salivary pH was measured using pH indicator strips because they are simple, rapid, economical, and clinically feasible. These strips contain pH-sensitive indicators that undergo a color change on contact with saliva, allowing an immediate estimation of pH using a standardized color scale. They are non-invasive, easy to handle, and convenient for use in clinical studies involving multiple participants. Although digital pH meters and electrodes provide greater analytical precision, pH indicator strips can serve as a practical chairside method when standardized procedures are followed, and the primary objective is to evaluate changes in pH over time. Previous studies have evaluated the clinical usefulness of pH paper-based methods and their agreement with electrometric techniques for salivary pH assessment [22,23].

The present study demonstrated different patterns of salivary pH among the three mouthrinse groups. A decreasing trend was observed in the chlorhexidine and Listerine groups, whereas Hetafu CUTE showed a statistically significant increase in salivary pH from baseline to one week. These findings suggest that the effect of mouthrinses on salivary pH may be formulation-dependent. This is supported by Tolentino et al., who evaluated salivary and tongue-coating pH before and after the use of different mouthrinses and reported differential changes in salivary pH, with some formulations increasing pH while others producing a decrease [24]. The observed differences may be attributed to variations in active ingredients and chemical composition, as well as differences in exposure duration and timing of pH assessment.

Milleman et al. evaluated 253 healthy adults and reported that salivary pH remained within the neutral range for the Listerine mouthrinse groups following a single use, with no lowering of salivary pH during the 30-minute assessment period. In contrast, the present study demonstrated a decreasing trend in salivary pH in the Listerine group over the study period [25]. This discrepancy could be explained by the difference in study populations and clinical conditions because Milleman et al. included healthy adults, while our study involved patients who received fixed orthodontic treatment. The fixed orthodontic appliances could affect plaque retention and the oral environment, which could modulate the salivary pH. This variation is further supported by the systematic review by Jakavičė and Žarovienė, which reported that unstimulated salivary pH generally decreases during fixed orthodontic treatment, although the available evidence remains heterogeneous [26].

Overall, the findings of the present study showed no statistically significant difference between the chlorhexidine and Listerine groups with respect to changes in salivary pH over time. Both groups demonstrated a gradual decrease in pH at the one-week assessment. In contrast, Hetafu CUTE was associated with a more favorable change in salivary pH compared with chlorhexidine and Listerine during the study period. However, the underlying mechanisms responsible for this observation cannot be established from the present data, as salivary buffering capacity and changes in the oral microbiome were not directly assessed. Nevertheless, maintenance of a favorable salivary environment may be clinically relevant in orthodontic patients, in whom prolonged plaque accumulation and acidogenic challenges can contribute to enamel demineralization. Considering these initial results, Hetafu CUTE mouthwash was associated with an increase in salivary pH in the present study. The described alkaline pH of Hetafu CUTE (7.62) may well be one contributing factor, although the relative contribution is unclear, and further studies are needed to elucidate this issue. Larger randomized trials are warranted that would include a wider follow-up, standardized physicochemical assessment, probiotic strain and viable-count evaluation, measurement of salivary buffering capacity, and microbiological analysis to understand the mechanisms responsible for the observed alterations in salivary pH.

Clinical implications

The maintenance of a favorable oral environment is of particular importance to patients who undergo orthodontic treatment with fixed appliances. An increase in the pH of saliva observed after the use of Hetafu CUTE in the current study indicates a possible beneficial effect of the product on the oral environment. A high pH level of 7.62 is most probably explained by the alkalinity of the product. Unfortunately, the current study did not assess enamel demineralization, formation of WSLs, remineralization, and caries development. Thus, the increased pH of saliva observed in the study cannot be considered an indicator of the prevention of WSLs. Further long-term investigations on caries development, salivary pH, buffering capacity, microbial activity, and possible adverse effects of the product are needed before any definite conclusions on the prevention of WSLs can be made.

Limitations and future directions

Notwithstanding the noteworthy results, several restrictions need to be taken into account. Salivary pH was assessed using pH strips, which may provide less accuracy than digital pH meters, and the study's duration was rather brief. In addition, the subjective color-matching nature of the strips and the absence of assessment of inter-observer or intra-observer measurement reliability may have introduced measurement variability. Moreover, other variables such as flow rate, microbial composition, and salivary buffering capacity were not assessed. To validate the findings and establish the treatment’s efficacy in the long run, further studies involving a larger sample size, prolonged follow-up periods, and comprehensive biochemical and microbiological assessments are recommended.

## Conclusions

Within the limitations of this study, the Hetafu CUTE mouthwash was found to be associated with a greater increase in the pH of the saliva over the study period compared to the chlorhexidine and Listerine mouthwashes among patients on a fixed orthodontic appliance. The latter two exhibited a decreasing trend in the pH of the saliva during the duration of this study. The reported alkaline pH of Hetafu CUTE (7.62) may account for this change, but the current study did not demonstrate that this effect was due to its alkalinity, botanical elements, probiotic components, or the combination thereof. Moreover, this study did not directly assess enamel demineralization or the development of WSLs. Therefore, this finding should not be construed to mean that the increase in the pH of the saliva would prevent the formation of WSLs. Large randomized controlled trials of superior duration with standardized measurements of pH, salivary buffering capacity, microbiological properties, and clinical assessment of enamel demineralization are necessary to fully elucidate the clinical significance of these results.
